# A Trilaminar-Catalytic Layered MEA Structure for a Passive Micro-Direct Methanol Fuel Cell

**DOI:** 10.3390/mi12040381

**Published:** 2021-04-01

**Authors:** Huichao Deng, Jiaxu Zhou, Yufeng Zhang

**Affiliations:** 1 School of Mechanical Engineering and Automation, Beihang University, Beijing 100191, China; denghuichao@buaa.edu.cn; 2 MEMS Center, Harbin Institute of Technology, Harbin 150001, China; yufeng_zhang@hit.edu.cn

**Keywords:** micro-direct methanol fuel cell, trilaminar-catalytic, water management, methanol crossover

## Abstract

A membrane electrode assembly (MEA) with a novel trilaminar-catalytic layered structure was designed and fabricated for a micro-direct methanol fuel cell (μ-DMFC). The trilaminar-catalytic layer comprises three porous layers. The medial layer has a lower porosity than the inner and outer layers. The simulation results predicted a lower water content and a higher oxygen concentration in the trilaminar-catalytic layer. The novel trilaminar-catalytic layer enhanced the back diffusion of water from the cathode to the anode, which reduces methanol crossover and improves oxygen mass transportation. The electrochemical results of the half-cell test indicate that the novel MEA has a greatly increased cathode polarization and a slightly increased anode polarization. Thus, this novel μ-DMFC structure has a higher power density and a longer discharging time, and hence may be used in portable systems.

## 1. Introduction

Due to the advantages conferred by their high energy density, facility of operations, fuel storage, and lack of pollution, proton exchange membrane fuel cells have been proposed as power sources for portable applications, automobiles, and stationary power plants [1,2,3]. The direct methanol fuel cell (DMFC) in the proton exchange membrane fuel cell provides greater advantages in portable applications and has attracted more attention in academia and industry [4,5,6]. This fuel cell uses liquid methanol as fuel. Compared with hydrogen, liquid methanol has many advantages, such as being easy to obtain, low cost, and having low storage and transport requirements. Many studies focused on the use of direct methanol fuel cells. A four-cell modular passive DMFC stack was designed, fabricated, and tested previously. A multi-module stack and DC–DC converter were combined to power an experimental fan and charge a mobile phone [7], while a passive direct methanol fuel cell, acting as transducer of an electrochemical sensor, was used to the detect of carcinoembryonic antigen [8]. Part of the reason for the success of these applications is that fuel cells provide several times more energy than batteries. With the increasing demand for portable energy, micro-direct methanol fuel cells (μ-DMFCs) are becoming a promising power source in the fuel cell family.

However, related research demonstrates that the performance of current µ-DMFCs is far below the expected theoretical values [9]. Thus, it is important to study the mass transfer characteristics, reaction mechanism, and characteristic parameters of these cells as it may help to improve our understanding of the internal mass transfer and the electrochemical reaction mechanism within µ-DMFCs. These studies will provide rationales for the optimization of the structure and performance of future µ-DMFCs. The crucial factor restricting the output performance of traditional µ-DMFCs is the low efficiency of the mass transport of oxygen at the cathode, often resulting from water flooding the cathode surface. At present, researchers are primarily focused on the water back diffusion from the cathode to the anode, as this can potentially solve the flooding at the cathode and decrease methanol crossover. A new hybrid catalyst layer (CL) was described in reference [10], wherein relatively hydrophilic and hydrophobic CLs were combined to form a hybrid catalyst layer. The relatively hydrophobic CLs were designed using hydrophobic binder polytetrafluoroethylene (PTFE), and the relatively hydrophilic CLs were designed using the hydrophilic binder Nafion.

Experimental results indicated that the hydrophilic and hydrophobic control of CLs can effectively produce better methanol and water concentration distribution. A new cathode micro-porous layer (MPL) was proposed with fluorinated carbon nanotubes [11]. The experiment results showed that the new microporous layer was free of cracks and had super hydrophobicity. Under the neat methanol operating conditions of passive direct methanol fuel cells, the water recovery flux significantly increased, improving the performance of the cell. A three-dimensional graphene framework was used for the first time to manufacture cathode MPL for enhancing water management [12]. The intrinsic pore structure and characteristics of the graphene frame can increase the hydraulic pressure of the cathode CLs, thus enhancing the water back diffusion from cathode to anode and improving the mass transfer of the cathode. The experimental results showed that the performance and stability of the prepared cells were improved remarkably. Reduced graphene oxide deposited in stainless steel fiber felt (SSFF) as a gas diffusion layer (GDL) was applied to a micro-direct methanol fuel cell [13]. This enhanced the back diffusion of water while reducing the anodic methanol crossover. The experimental results showed that the cell at 4 M and 5 M methanol concentration with the new diffusion layer performed better than a conventional cell.

From the literature review, water back diffusion enhancement is an effective method for water management, preventing flooding in the cathode and lowering the methanol crossover from the anode to the cathode. Although significant advancements have been achieved using the aforementioned methods, the oxygen mass transport within the catalyst layer is still a problem. In this study, we examined whether a trilaminar-catalytic layered membrane electrode assembly (MEA) structure could facilitate the increase in water back diffusion and promote oxygen mass transportation. The trilaminar-catalytic layer comprises an inner, medial, and outer layer. The medial layer has lower porosity than the inner and outer layers. This creates a water pressure gradient between the inner and medial layers, and an oxygen concentration gradient between the outer and medial layers. Thus, the trilaminar-catalytic layered MEA can enhance water back diffusion from the cathode to the anode, as well as oxygen mass transportation, by creating beneficial gradients.

## 2. Simulation Analysis

**Figure 1:**
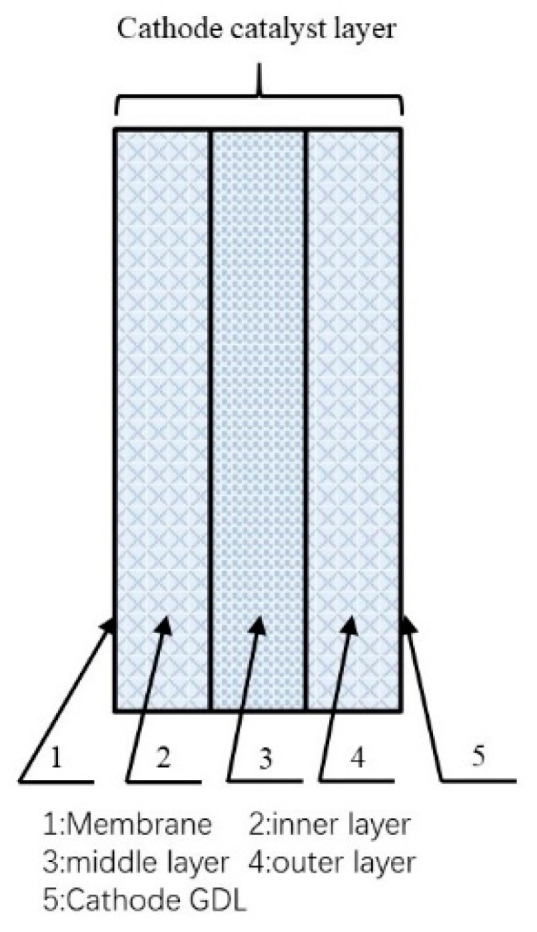
The modeling domain.

To effectively validate the trilaminar-catalytic layered cathode structure, a three-dimensional static model was characterized and simulated using a two-phase thermal model for a passive DMFC [14,15]. For simplification and improved results, the region simulated included three cathode catalyst layers, each with a different porosity. The porosity of middle layer was set to 0.4 [16,17] and the porosity of the other layers was set to 0.7. The simplified two-dimensional modeling domain of the DMFC is shown in Figure 1. This model was simulated under the following simplifications and assumptions:

(1) The fuel cell works under steady-state and isothermal conditions.
(2) The fuel cell and environment have thermal isolation so that the heat does not disperse from the gas diffusion layer (GDL) into the environment.
(3) The concentration change in methanol does not influence the cathode.
(4) The boundary condition of the left line is Nafion membrane, and the boundary condition of the right line is cathode GDL.

The coagulum model was used to describe the electrode reaction of the catalyst layer. The coagulum included the catalyst and the embedded polymer electrode carbon atoms. This model equation evolved from the analytical solutions for spherical porous particle diffusion reactions. The potential distribution under a DC conductive medium is described as follows:

(1) $$\nabla \cdot \left(-\kappa_{m,eff}\nabla \phi_{m}\right)=0,$$

(2) $$\nabla \cdot \left(-\kappa_{s,eff}\nabla \phi_{s}\right)=0,$$

where $\phi_{s}$ is the potential of the membrane, $\phi_{m}$ is the potential of the cathode, $\kappa_{s,eff}$ is the solid effective electronic conductivity, and $\kappa_{m,eff}$ is the membrane ionic conductivity. The coagulum model indicated the current density of the catalytic layer, which comprised ion conductive materials and parts attached to the catalyst’s electronic conductive particles. The coagulum, which had a constant electronic and ionic potential, was analytically expressed as the local current density, through the diffusion kinetics equation and through the kinetic equation of the electrode process (Butler–Volmer equation) [18]. The cathode current density equation was formulated as follows:

(3) $$j_{agg,c}=-6n_{c}F\left(\frac{D_{agg}}{R_{agg}^{2}}\right)\left(1-\lambda_{c}coth\lambda_{c}\right)\beta_{c},$$

(4) $$\;\;\;\;\;\;\;\lambda_{c}=\sqrt{\frac{i_{0c}SR_{agg}^{2}}{4Fc_{O_{2},ref}D_{agg}}\exp\left(-\frac{F}{2RT}\eta_{c}\right)},$$

(5) $$\beta_{c}=c_{O_{2},agg},$$

(6) $$\eta_{c}=\phi_{s}-\phi_{m}-E_{eq,c},$$

where $D_{agg}$ is the coagulum gas diffusion coefficient (m^2^/s), $R_{agg}$ is the coagulum radius, $n_{c}$ is the charge transfer coefficient (which is −2), S is the specific area of catalytic material in coagulum, F is the Faraday constant (C/mol), R is the gas constant, T is the temperature (K), $c_{i,ref}$ is the reference substance concentration (mol/m^3^), $c_{i,agg}$ is the coagulum surface corresponding concentration (mol/m^3^), $i_{0c}$ is the exchange current density (A/ m^2^), and $E_{eq,c}$ is the balance voltage.

The reactive gas was considered to be an ideal gas transferred by diffusion and convection, while the electrode was assumed to be an isotropic porous medium with uniform morphological characteristics, such as porosity and permeability. The gas in the electrode was a continuous phase, so Darcy’s law proved that the pressure gradient, the viscosity of the fluid, and the porous medium structure determined the water velocity, $u_{l}$, within the porous medium:

(7) $$u_{l}=-\frac{Kk_{rl}}{\mu_{l}}\nabla p_{l},$$

where K is the absolute permeability of the porous medium, $k_{rl}$ is the relative permeability of the liquid phase, $u_{l}$ is the dynamic viscosity of the liquid phase, and $p_{l}$ is the pressure of the liquid phase. In the cathode boundary, the electrochemical reaction rate and gas flow velocity were calculated from the total mass flow, according to Equation (8).

(8) $$-n\cdot {u|}_{cathode}=-\frac{j_{cathode}}{\rho F}\left[\frac{M_{O_{2}}}{4}+\left(\frac{1}{2}+\lambda_{H_{2}O}\right)M_{H_{2}O}\right],$$

where${\;\lambda }_{H_{2}O\;}$is the electro-osmotic drag coefficient of water; the above model considers the three substances in the cathode: oxygen, water, and nitrogen.

The Maxwell–Stefan multicomponent diffusion was determined by the following Equation (9):

(9) $$\frac{\partial }{\partial t}\rho \omega_{i}+\nabla \cdot \left[-\rho \omega_{i}\overset{N}{\underset{j=1}{\sum }}D_{ij}\left\{\frac{M}{M_{j}}\left(\nabla \omega_{j}+\omega_{j}\frac{\nabla M}{M}\right)+\left(x_{j}-\omega_{j}\right)\frac{\nabla p}{p}\right\}+\omega_{i}\rho u+D_{i}^{T}\frac{\nabla T}{T}\right]=R_{i}.$$

The software calculated the mass fraction of the solution $\omega_{i}$. The proton exchange membrane fuel cell model assumed that the temperature-driven diffusion was not obvious, and that the source term R was set to 0. For the three types of gases in cathode (oxygen indicated by 1, water by 2, and nitrogen by 3), the mass transfer could be simulated using the following equations, which determined the flow rate together with Darcy’s law:

(10) $$\nabla \cdot \{-\rho \omega_{1}\underset{j}{\sum }[D_{1j}>(\nabla x_{j}+\left(x_{j}-\omega_{j}\right)\frac{\nabla p}{p}>)]\}=-\left(\rho u\cdot \nabla \omega_{1}\right),$$

(11) $$\nabla \cdot \{-\rho \omega_{2}\underset{j}{\sum }[D_{1j}>(\nabla x_{j}+\left(x_{j}-\omega_{j}\right)\frac{\nabla p}{p}>)]\}=-\left(\rho u\cdot \nabla \omega_{2}\right),$$

(12) $$\omega_{3}=1-\omega_{1}-\omega_{2}$$

where p is the pressure (Pa), T is the temperature (K), and u is the velocity (m/s). At the junction of the electrode and the membrane, the mass flux of oxygen and water in the cathode is determined by the electrochemical reaction rate:

(13) $$-n\cdot {N_{O_{2}}|}_{cathode}=-\frac{j_{cathode}}{4F}M_{O_{2}},$$

(14) $$-n\cdot {N_{H_{2}O}|}_{cathode}=-\frac{j_{cathode}}{F}(\frac{1}{2}+\lambda_{H_{2}O})M_{H_{2O}}.$$

**Figure 2:**
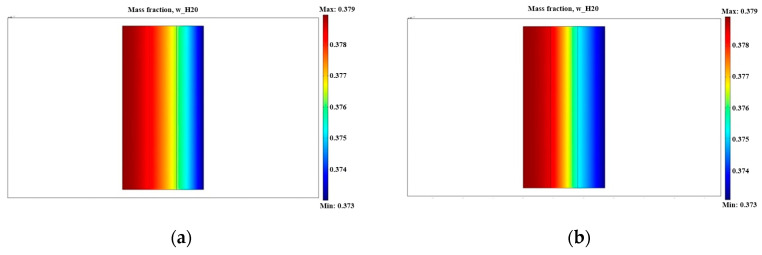
Comparison of water mass fraction in the traditional catalyst layer and trilaminar-catalytic layer: (**a**) traditional catalyst layer; (**b**) trilaminar-catalytic layer.

The coupled equations were numerically calculated by the finite element method using COMSOL Multiphysics 5.5. We used a physics-controlled mesh to automate meshing. The finer element size was selected reasonably, and the number of elements was 11,466. The temperature over the entire solution domain was set at a constant value of 298 K. Figure 2 shows the comparison of water mass fraction between the traditional catalyst layer and the trilaminar-catalytic layer. The traditional catalyst layer was divided into three layers, all of which had the same porosity. The trilaminar-catalytic layer comprised the inner, medial, and outer layers. In both cases, the water mass fraction of the catalyst layer remained between 37% and 38%. The water was not only generated by the chemical reaction in the catalyst layer, but also penetrated from the anode through the proton exchange membrane. The water content increased from the outer to the inner catalytic layers, which was similar to, yet more pronounced than, the behavior at the proton exchange membrane. Too much water content at the cathode inhibited the reaction and resulted in the flooding of the cathode, which was detrimental to the stability of the battery output performance, and thus may have lowered the long-term output performance. However, the reaction at the anode required the water’s participation, so it was more beneficial to allow water back diffusion from the cathode to the anode, and to use the proton exchange membrane to improve the performance of the μ-DMFC.

According to the simulation results, the water content in the inner layer of the trilaminar-catalytic layer was higher than that of the traditional catalytic layer. Furthermore, the water content in the middle and outer layers of the trilaminar-catalytic layer were lower than that of the traditional catalytic layer. This was due to the variation in porosity. In the trilaminar-catalytic layer, the porosity of the inner layer was 0.7, which is higher than that of the middle layer, at 0.4. The water diffusion was blocked by the middle layer, which increased the hydraulic pressure in the catalyst layer and improved the water back diffusion from the cathode to anode. The porosity of the middle layer when compared to the outer layer was lower. Therefore, the water generated by the reaction was more easily discharged through the pores of the outer layer. Due to the lower water pressure, the gas diffusion mass transfer ability improved and the cell output performance increased.

**Figure 3:**
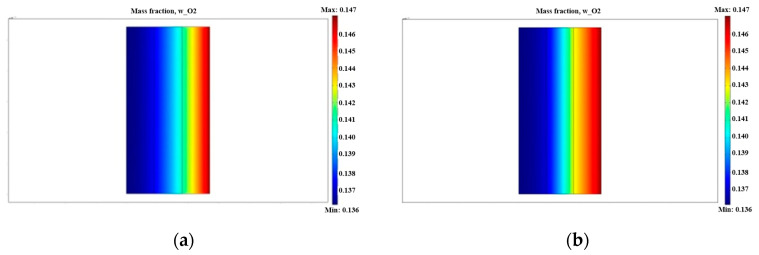
Comparison of oxygen mass fraction in the traditional catalyst layer and trilaminar-catalytic layer: (**a**) traditional catalyst layer; (**b**) trilaminar-catalytic layer.

Figure 3 compares the oxygen mass fraction in the traditional catalyst layer with that of the trilaminar-catalyst layer. As can be seen from the results of these two simulations, the oxygen mass fraction in the middle and outer layers of the trilaminar-catalytic layer was higher than that of the traditional catalytic layer. From the outer layer to the inner layer, the oxygen content and the reaction rate decreased gradually. When comparing these two conditions, the structure of the trilaminar-catalytic layer, with its differing porosity, produced superior performance. As the outer porosity of the catalyst layer was larger (0.7), gas transfer was easier, which resulted in more oxygen reaching the middle layer of the catalyst layer, promoting the chemical reaction.

## 3. Fabrication and Assembly Results

**Figure 4:**
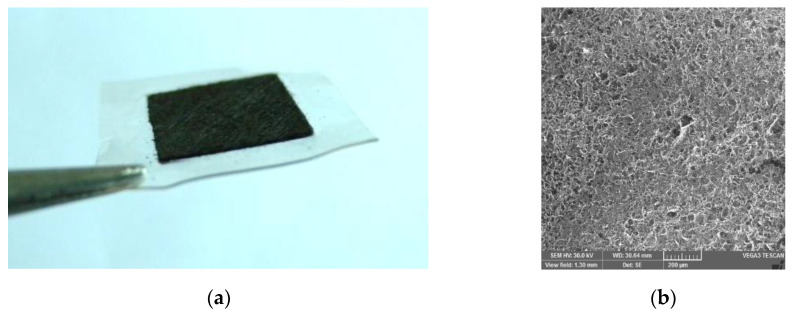
Picture of the passive micro-direct methanol fuel cell (μ-DMFC) with the trilaminar-catalytic layer structure: (**a**) Photograph of the membrane electrode assembly (MEA) with the trilaminar-catalytic layer; (**b**) partial pore former (PF) cathode scanning electron microscope image (SEM).

A mixture of 90 wt % XC-72 and 10 wt % PTFE was sprayed onto the surface of carbon paper that served as a microporous layer, with a carbon loading of 4 mg cm^−2^. The anode catalyst inks (comprising 40 wt % Pt-Ru/C, 5 wt % solubilized Nafion, isopropanol, and deionized water in 1:1 (v:v) were uniformly sprayed using a spray gun onto the GDL with a catalyst loading of approximately 8.33 mg cm^−2^. Cathode catalyst ink A comprised 40 wt % Pt/C, 5 wt % solubilized Nafion, isopropanol and deionized water in 1:1 (v:v). Cathode catalyst ink B was prepared by mixing 25 wt % pore-former (PF) NH_4_HCO_3_ into ink A. The cathode gas diffusion electrodes (GDEs) were made by separately spraying ink A and ink B over the microporous treated carbon paper, with a catalyst loading of 6.25 mg cm^−2^; these are referred to as the no PF cathode and the full PF cathode. The trilaminar-catalytic layered cathode GDE, called the partial PF cathode, was constructed by spraying ink B over the microporous treated carbon paper with a catalyst loading of approximately 2.09 mg cm^−2^ (outer layer), then by spraying ink A, increasing catalyst loading to approximately 4.18 mg cm^−2^ (middle layer), and finally by spraying ink B, increasing catalyst loading to approximately 6.25 mg cm^−2^ (inner layer). The Nafion membrane was pretreated in water baths of deionized water, 3 wt % H_2_O_2_, and 3 wt % H_2_SO_4_ for 1 h at 353 K. The Nafion membrane was sandwiched between two GDEs and hot pressed under 4 MPa at 303 K for 120 s to fabricate a 5-layer MEA with an active area of 1.15 × 1.15 cm. Thus, the MEA was completely assembled as shown in Figure 4a. The partial PF cathode scanning electron microscope image (SEM) [19,20,21] is shown in Figure 4b.

**Figure 5:**
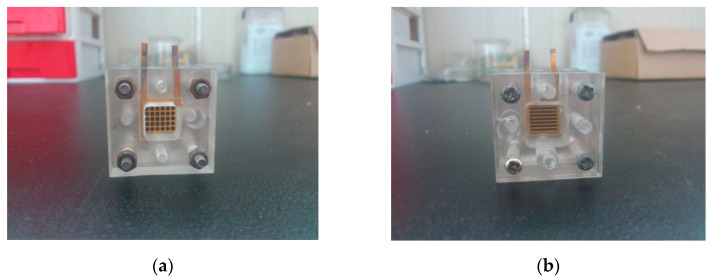
Photograph of the assembled μ-DMFC: (**a**) photograph of the MEA with trilaminar-catalytic layer; (**b**) partial PF cathode scanning electron microscope image (SEM).

The passive μ-DMFC had anode parallel channels [22] with an open ratio of 50%. The microchannel had a width equal to 0.83 mm, and the channel length was equal to 10 mm. The cathode current collector had self-breathing openings with an open ratio of 37%. The hole diameter was 1.32 mm. Stainless steel plates with a thickness of 300 μm were chosen to fabricate the anode and cathode current collectors. A 500 nm layer of Au was deposited on the current collectors by a magnetron sputtering ion plating (MSIP) to reduce contact resistance and to avoid chemical corrosion. To complete the assembly, two fixtures with current collectors and the other components were clamped together using four screws. Outside the anode collector plate was a 2.5 mL methanol solution storage chamber. The picture of the passive μ-DMFC after assembly is shown in Figure 5.

## 4. Result and Discussion

To analyze the influence of the trilaminar-catalytic layered cathode on the performance of μ-DMFC, the experiments were passively fed with a 2.5 mL methanol reservoir that was filled with dilute methanol and tested at 298 K. Before operation, the MEA needed an activation process to achieve optimal performance. First, deionized water was poured in the methanol reservoir to humidify the MEA, while the cell was kept at 333 K for 3 h. Second, to activate the catalysts, a 2.0 M methanol solution was fed into the methanol reservoir, while the cell was kept at 333 K. Under these conditions, the cell was operated at 100 mA for 4 h.

**Figure 6:**
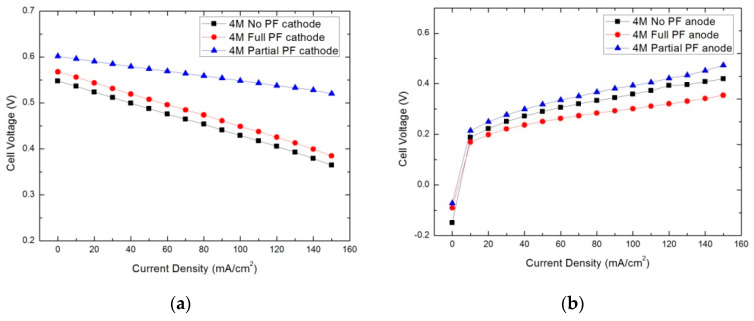
Cathode and anode potential of no PF cathode, full PF cathode, and partial PF cathode with 4 M methanol solutions at 298 K: (**a**) cathode potential; (**b**) anode potential.

To further investigate the reaction inside the cell, a half-cell measurement was carried out [23,24]. A reliable half-cell measurement system requires a reference electrode with a fixed potential and a stable electrical contact. The reference electrode used in this paper was a 2.6 V Ag/AgCl electrode. Figure 6 shows the cathode and anode potentials for the no PF cathode, the full PF cathode, and the partial PF cathode, using a 4 M methanol solution at 298 K. Figure 6a shows that the partial PF cathode, which had its inner and outer layers mixed with the pore forming agent, demonstrated the highest potential. The full PF cathode, which had its whole catalytic layer mixed with the pore forming agent, performed medially. Finally, the no PF cathode, which had no contact with the pore-forming agent, had the lowest potential. In all cases, as the discharge current increased, the cathode potential gradually decreased. However, the cathode potential of the partial PF cathode, with the three-layered structure, decreased more slowly than the other two monolayer cathodes. The whole cell voltage is the sum of the potential difference between the anode and cathode. A higher cathode potential or a lower anode potential indicates a smaller electrode polarization, which improves the output voltage and the performance of the cell. Methanol exposure had a definite effect on the cathode potential. Methanol solutions of high concentration can lead to severe methanol permeability; this forms a mixed potential, causing a cathode potential drop and eventually exacerbating cathode polarization. Therefore, the decrease in the cathode potential of the partial PF cathode confirmed that the partial PF cathode increased water back diffusion via a hydraulic pressure gradient, thus reducing methanol crossover. The partial PF cathode enhanced the oxygen mass transport, which also improved cathode potential. Although the methanol crossover of the full PF cathode occurred in a more significant manner than that of the no PF cathode, the cathode potential of the full PF cathode was higher than that of the no PF cathode. This increase in cathode potential was due to the improved oxygen mass transport resulting from the porosity structure of the full PF cathode.

Figure 6b shows that the partial PF cathode had a higher anode potential than the other two cathode structures. This can be attributed to the lower temperature of the MEA in the partial PF cathode. The methanol crossover from anode to cathode was directly oxidized by oxygen and released a significant amount of heat. The anode reaction of methanol oxidation had a large activation polarization; however, the methanol crossover generated sufficient heat to decrease the anodic polarization. Therefore, the anodic polarization decreased with methanol crossover. This result suggested that the methanol crossover was reduced in the partial PF cathode by water back diffusion, and that the full PF cathode had the most pronounced methanol crossover.

**Figure 7:**
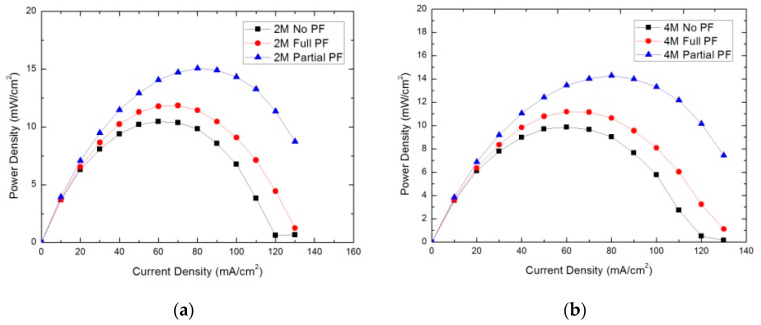
Power density of the μ-DMFCs with no PF cathode, full PF cathode, and partial PF cathode: (**a**) 2 M and (**b**) 4 M methanol solutions.

The performance of cells with three cathodes was measured by the constant current method. Figure 7 shows power density of the no PF cathode, the full PF cathode, and the partial PF cathode operating with 2 and 4 M methanol solutions at 298 K. It can be seen from the curve that the partial PF cathode had the best cell performance, the full PF cathode performed medially, and the no PF cathode had the poorest performance. When the discharge current increased, the differences in performance became more pronounced. When using the 2 M methanol solution, the partial PF cathode, discharged at an 80 mA/cm^2^ current density, reached a maximum power density of 22 mWcm^−2^, while the peak power densities of the full PF cathode and the no PF cathode were only 17 and 15 mWcm^−2^, respectively. When operating using the 4 M methanol solution, the maximal power densities decreased to 19, 12, and 9 mWcm^−2^, respectively. This is probably due to the increase in the methanol solution concentration, leading to the conclusion that methanol permeability is critical to performance. Therefore, the cathode polarization phenomenon was significant, and the cathode potential decreased, suggesting that the cathode polarization phenomenon causes output performance degradation. Moreover, we observed that the reduced power densities of the full PF cathode and the no PF cathode were much more obvious than that of the partial PF cathode. This proved that in the high concentration methanol solution, the methanol crossover in the partial PF cathode was not a serious detriment to the water back diffusion, while the other two cathodes suffered from severe methanol crossover. Therefore, the passive μ-DMFC of the partial PF cathode had the best performance.

**Figure 8:**
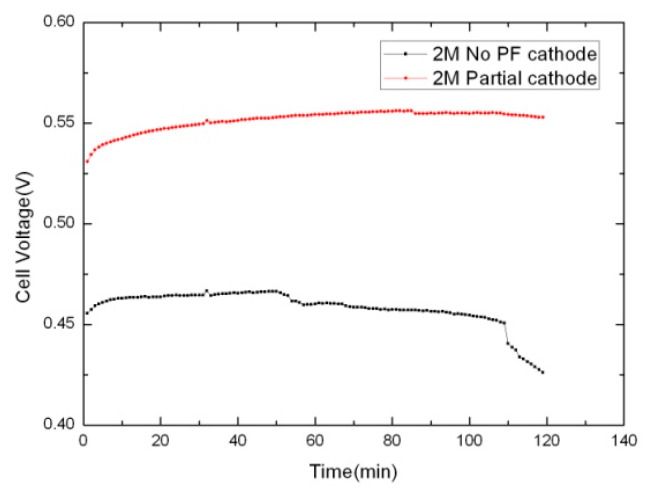
The cathode potential of no PF cathode and partial PF cathode under 80 mA cm^−2^ with 2 M methanol solution.

Figure 8 shows the cathode potential of the no PF cathode and the partial PF cathode discharging under 80 mA cm^−2^ with 2 M methanol at 298 K. The total discharge time was 120 min, and data were recorded every 1 min. The partial PF cathode structure caused the cathode potential to stay steady at approximately 0.53–0.55 V. The cathode potential of the no PF cathode began at approximately 0.46 V. As the reaction proceeded, the cathode potential decreased significantly after 100 min due to the water generated by the reaction not being discharged in time, causing flooding in the cathode. This phenomenon blocked the oxygen mass transportation and exacerbated the cathode polarization, which resulted in a reduction in the cathode potential. Therefore, it can be seen that the trilaminar-catalytic layered cathode can prevent flooding in the cathode, thus improving the output performance of the passive μ-DMFC.

To ascertain the water back diffusion effect of the trilaminar-catalytic layered cathode, the water transport coefficient was calculated by long-term charging. Table 1 shows the calculated water transport coefficients of the no PF cathode and the partial PF cathode. The water transport coefficient of the no PF cathode was 1.56, while the water transport coefficient of the partial PF cathode was less, only 1.05. The differences in the water transportation coefficients prove that the proposed trilaminar-catalytic layered cathode structure can effectively reduce the cathode water accumulation and slow down the cathode flooding phenomenon, thereby improving the output performance and stability of the passive μ-DMFC.

## 5. Conclusions

A passive μ-DMFC with a novel trilaminar-catalytic layered cathode performed better than one with a traditional catalyst layer due to the porosity gradient created in the trilaminar-catalytic layer. This gradient improved water back diffusion and oxygen mass transportation, both of which improved the overall μ-DMFC performance and enabled the partial PF cathode to obtain the optimal performance of 27 and 22 mWcm^−2^ at the concentrations of 2 M and 4 M methanol, respectively. This performance is higher than the no PF cathode and full PF cathode. The water back diffusion effect reduced methanol crossover and flooding in the cathode, which increased the cathode potential. Compared with the no PF cathode, the partial PF cathode structure was more stable, as the cathode potential remained steady at approximately 0.53–0.55 V and discharged under 80 mA cm^−2^ with 2 M methanol at 298 K. In addition, the water transfer coefficient of the partial PF cathode was 1.05, lower than that of the no PF cathode at 1.56, which also proves that the novel trilaminar-catalytic layered cathode effectively reduces cathode water accumulation and slows the cathode flooding phenomenon.

## Figures and Tables

**Table 1:** Water transport coefficient of no PF cathode and partial PF cathode.

	No PF	Partial PF
Final volume (mL)	1.90	2.00
Final concentration (wt %)	4.77	3.81
Water transport coefficient α	1.56	1.05

## Data Availability

Not applicable.
